# Transferrin-modified liposomes enhance chemosensitivity in hepatocellular carcinoma by suppressing RDM1-mediated DNA repair

**DOI:** 10.3389/fonc.2026.1792167

**Published:** 2026-03-23

**Authors:** Xiaoni Cai, Xiang Wang, Qiongdan Zhang, Fan Gao, Song Xu, Wenqiao Lyu, Jiayuan Ye, Fei Liu, Luting Zhang

**Affiliations:** 1 Department of General Surgery, Shangyu People’s Hospital of Shaoxing, Shaoxing, Zhejiang, China; 2 School of Medicine, Shaoxing University, Shaoxing, Zhejiang, China; 3 Department of Hepatobiliary Surgery, Shandong Provincial Third Hospital, Shandong University, Jinan, Shandong, China; 4 Shandong Provincial Third Hospital Medical Research Center, Hepatobiliary Minimally Invasive Research Lab, Jinan, Shandong, China; 5 Department of The First Clinical Medical School, Hainan Medical University, Haikou, Hainan, China; 6 Hainan Academy of Medicine Sciences, Haikou, Hainan, China; 7 Department of Infectious Diseases, Shangyu People’s Hospital of Shaoxing, Shaoxing, Zhejiang, China; 8 Department of Hepatobiliary Surgery, The First People’s Hospital of Lianyungang, Lianyungang, Jiangsu, China; 9 Department of Hepatobiliary Surgery, Lianyungang Clinical College of Nanjing Medical University, Lianyungang, Jiangsu, China; 10 Department of Hepatobiliary Surgery, The First Affiliated Hospital of Kangda College of Nanjing Medical University, Lianyungang, Jiangsu, China

**Keywords:** chemosensitization, hepatocellular carcinoma, liposome, RDM1, transferrin

## Abstract

**Introduction:**

RDM1 is linked to poor prognosis in hepatocellular carcinoma (HCC) chemotherapy. We investigated its post-transcriptional regulation and developed a targeted co-delivery strategy to enhance chemosensitivity.

**Methods:**

Clinical data were analyzed for RDM1 prognostic value. Post-transcriptional regulation by 5-azacytidine (5-Aza) and synergy with doxorubicin (ADM) were assessed via DNA damage repair and apoptosis assays. A transferrin-modified liposome (AA@Tf-Lip) was constructed for co-delivery, and antitumor efficacy evaluated *in vitro* and *in vivo*.

**Results:**

High RDM1 expression predicted poor HCC survival. 5-Aza downregulated RDM1 by reducing mRNA stability, inhibiting RAD51-mediated homologous recombination repair, and synergistically activating p53/Bax apoptosis with ADM. AA@Tf-Lip enhanced cellular uptake and tumor specificity, achieving higher tumor inhibition and reduced cardiotoxicity versus free drugs.

**Conclusion:**

Indirect RDM1 inhibition via post-transcriptional regulation combined with targeted co-delivery effectively enhances HCC chemosensitivity, offering a safe, precise therapeutic strategy.

## 1 Introduction

Hepatocellular carcinoma (HCC) is one of the leading causes of cancer-related deaths worldwide, with a 5-year survival rate of <20% (1). Although anthracycline chemotherapeutics represented by doxorubicin (ADM) are widely used in advanced HCC treatment, the insufficient chemosensitivity of tumor cells severely limits clinical efficacy. Studies have shown that improving chemosensitivity is a key strategy to enhance the clinical efficacy of ADM in HCC treatment, and the abnormal activation of DNA repair pathways is a major factor leading to low chemosensitivity (2). Among which homologous recombination repair (HRR) is a key mechanism for cancer cells to escape DNA damage (3). However, current small-molecule drugs targeting DNA repair pathways still face bottlenecks such as insufficient specificity and high systemic toxicity, highlighting the urgent need to explore new molecular intervention targets and precise regulatory strategies (4).

As a core molecule in HRR, the oncogenic effect of RAD52 motif-containing 1 (RDM1) has been validated in multiple cancer types: in papillary thyroid carcinoma (5), lung adenocarcinoma (6), and breast cancer (7), high RDM1 expression drives malignant proliferation of tumor cells, while RDM1 knockdown significantly induces cell cycle arrest and activates apoptotic signals. More notably, Guo et al. found that RDM1 promotes neuroblastoma growth by activating the RAS-Raf-MEK-ERK signaling axis (8). This suggests that while RDM1 plays a core role in DNA repair, changes in its expression level or activity may further influence tumor progression by regulating other key signaling pathways (such as the RAS pathway). Although it remains to be explored in depth whether the activation of these “non-classical” pathways partially depends on alterations in its DNA repair function, it undoubtedly reveals the complexity of RDM1’s function and its multi-dimensional role in tumor initiation and development. Significantly, as a typical HRR-deficient tumor (9), abnormal DNA damage repair in the HCC tumor microenvironment is closely associated with poor prognosis, while the specific role of RDM1 in this process remains unclear.

Insufficient chemosensitivity caused by compensatory activation of DNA repair pathways is a core barrier for ADM treatment in HCC. Recent studies have revealed that epigenetic mechanisms can regulate chemotherapy response by dynamically remodeling the expression profile of DNA repair genes—for example, in BRCA1-mutated breast cancer, DNMT1-mediated promoter hypermethylation can silence the RAD51 gene, induce HRR deficiency, and sensitize cells to PARP inhibitors (10). Inspired by this, combined with the research gap of no widely validated specific small-molecule inhibitors for RDM1, this study adopted an epigenetic intervention strategy (e.g., DNMT1 inhibition) to systematically evaluate the function and druggability of RDM1 in HCC, aiming to fill the existing research gap.

Previous studies have confirmed that 5-azacytidine (5-Aza) can indirectly regulate RDM1 expression and function by inhibiting the DNA methylation activity of DNMT1. However, our current study confirmed that 5-Aza does not regulate RDM1 through the classical DNA demethylation-mediated transcriptional activation pathway, but instead downregulates RDM1 expression via post-transcriptional mechanisms by reducing the stability of RDM1 mRNA (11, 12). Based on this, this study focused on this DNA methyltransferase inhibitor and hypothesized that it can downregulate RDM1 expression via post-transcriptional regulation, block the HRR pathway, and thereby enhance the DNA-damaging effect of ADM to improve chemosensitivity. However, both 5-Aza and ADM have systemic toxicity (e.g., cardiac injury, myelosuppression), which may limit their clinical application (13, 14). It is worth noting that transferrin receptor (TfR) is highly expressed on the surface of hepatocellular carcinoma (HCC) cell membranes but lowly expressed in normal liver tissues (15), which provides an ideal target for active targeted delivery systems. Therefore, building on the distinct expression pattern of TfR in HCC, this study developed a TfR targeted controlled release nanosystem. By leveraging the synergistic effects of epigenetic-mediated chemosensitization and chemotherapeutic killing, we aim to mitigate the off target toxicity commonly associated with small molecule drugs, thereby offering a precise therapeutic strategy to enhance chemosensitivity in HCC.

## 2 Materials and methods

### 2.1 Materials and animals

5-Azacytidine and 2-iminothiolane hydrochloride were purchased from Shanghai Macklin Biochemical Technology Co., Ltd.; doxorubicin hydrochloride was obtained from Shanghai Aladdin Biochemical Technology Co., Ltd.; CCK-8 and 0.1% crystal violet were from Wuhan Servicebio Technology Co., Ltd.; antibodies against RDM1, γH2AX RAD51 and Alexa Fluor 594-conjugated secondary antibody were from Hangzhou Huaan Biotechnology Co., Ltd.; DSPE-PEG-MAL (2000) was from GuangZhou Tanshtech Co., Ltd.; cholesterol and egg yolk lecithin were from Beijing Solarbio Science & Technology Co., Ltd.; Transferrin was from Shanghai Acmec Biochemical Technology Co., Ltd.; ultrafiltration tubes were from Anhui Baisha Biotechnology Co., Ltd.; and the apoptosis detection kit was from Shanghai Beyotime Biotechnology Co., Ltd.

Nude Balb/C mice (18–22 g) were supplied by Beijing Vital River Laboratory Animal Technology Co., Ltd. (Beijing, China). Animal procedures were in accordance with the Guide for the Care and Use of Laboratory Animalspublished by the National Institutes of Health and were approved by the Experimental Animal Welfare and Ethics Committee of Shandong Provincial Third Hospital (No. SYDW-2025052).

### 2.2 Instruments

Microplate reader (BioTek Synergy 2), flow cytometer (BD FACSCalibur), transmission electron microscope (TEM, JEOL JEM-1010), ultraviolet-visible spectrophotometer (Shimadzu UV-1800), fluorescence spectrophotometer (Hitachi F-7000), confocal laser scanning microscope (CLSM, Olympus SV200), and digital slide scanner (Leica Aperio CS2).

### 2.3 Methods

#### 2.3.1 Bioinformatics analysis

RDM1 mRNA expression data and corresponding clinical follow-up information of HCC patients were retrieved from the GEPIA database (http://gepia.cancer-pku.cn/) for prognostic analysis. Meanwhile, the expression levels of RDM1 and candidate interacting proteins in normal liver tissues were obtained and imported into the DAVID database for survival curve analysis and “GO Biological Process (BP)” analysis. The human “RDM1” gene name was input into the STRING database (https://cn.string-db.org/) to construct a protein-protein interaction (PPI) network dataset, which was then visualized using Cytoscape.

#### 2.3.2 Cytotoxicity assay

HepG2, Huh7 or HUVEC cells in the logarithmic growth phase were digested, adjusted to 5×10³ cells/well, and seeded into 96-well plates (100 μL/well). Cells were pre-incubated at 37 °C in a 5% CO_2_ incubator for 24 h to allow adherence. After adherence, the original medium was aspirated, and fresh medium containing different concentrations of test drugs was added, followed by further incubation for 24 h. After incubation, the drug-containing medium was removed, and 100 μL of basic medium with 10 μL CCK-8 reagent was added to each well. Plates were gently shaken and incubated in the dark for 1 h. Finally, absorbance (OD value) at 450 nm was measured using a microplate reader to calculate cell viability. The IC_50_ value was analyzed via dose-response curves, and CompuSyn software was used to calculate the combination index (CI) and plot the Fa-CI curve. siRNA-mediated RDM1 knockdown experiment: HepG2 cells were transfected with RDM1-specific siRNA or negative control siRNA, and the knockdown efficiency was verified by qPCR and Western blot; the chemosensitivity of transfected cells to ADM was detected by CCK-8 assay.

#### 2.3.3 Colony formation assay

HepG2 and Huh7 cells in the logarithmic growth phase were digested, counted, and adjusted to 500 cells/mL with complete medium. Then, 2 mL of cell suspension (≈1000 cells/well) was added to 6-well plates. Plates were gently shaken to evenly distribute cells and incubated statically at 37 °C in a 5% CO_2_ incubator for 7–14 days (fresh drug-containing medium was replaced every 2–3 days). After incubation, the medium was discarded, and cells were gently washed 1–2 times with PBS. Cells were fixed with 4% paraformaldehyde for 15 min and stained with 0.1% crystal violet for 10 min. Excess dye was removed by rinsing with running water, and plates were air-dried at room temperature (RT). Cell colonies were counted to calculate the colony formation rate.

#### 2.3.4 Quantitative polymerase chain reaction

RNA samples were prepared according to the instructions provided with the total cellular RNA extraction kit. The concentration of RNA was measured using a Qubit 4.0, and agarose gel electrophoresis was employed to assess RNA integrity. The extracted RNA was reverse transcribed using a reverse transcription kit, and quantitative PCR experiments were conducted with the AceQ qPCR SYBR Green Master Mix (without ROX). The results were obtained online, and the relative expression of target genes was calculated and statistically analyzed. Primer sequences for RDM1, TP53(p53), and BAXwere synthesized by Zhenke Biotechnology (sequences in **Supplementary Table 1**). mRNA stability assay: HepG2 cells were treated with 5-Aza, and then actinomycin D was added to block nascent transcription; the expression of RDM1 mRNA at different time points was detected by qPCR to calculate the mRNA half-life. MSP assay: Genomic DNA of HepG2 cells before and after 5-Aza treatment was extracted, and the methylation status of RDM1 promoter was detected by MSP according to the kit instructions.

#### 2.3.5 Immunofluorescence assay

HepG2 cells grown on coverslips and treated with ADM (1 μM) and 5-Aza (3 μM) were fixed with 4% paraformaldehyde for 15 min, washed with PBS, permeabilized with 0.1% Triton X-100 for 10 min, and blocked with 5% BSA for 1 h. γH2AX staining: cells were incubated with anti-γH2AX primary antibody at 4 °C for 2 h, washed with PBS, and incubated with fluorescent secondary antibody (Alexa Fluor 594) in the dark for 1 h. Nuclei were stained with DAPI for 5 min, and coverslips were mounted with anti-fluorescence quenching medium. ImageJ software was used for quantitative analysis of γH2AX foci fluorescence intensity.

#### 2.3.6 Preparation and characterization of Tf-modified drug-loaded liposomes

Egg yolk lecithin (72 mg), cholesterol (30 mg), and DSPE-PEG-MAL (2000, 10 mg) were dissolved in chloroform. A lipid film was formed via rotary evaporation at 40 °C and vacuum-dried. The lipid film was hydrated with 10 mL pH 7.4 PBS containing 5-Aza (15 mg) and ADM (5 mg) at 40 °C for 1 h. After vortex oscillation, the mixture was extruded through a 200 nm membrane to obtain drug-loaded liposomes. Separately, Tf (20 mg) was reacted with 2-iminothiolane hydrochloride (1.5 mg) at RT for 1 h to generate thiolated Tf, which was purified via ultrafiltration and conjugated with liposomes (containing DSPE-PEG-MAL) at 4 °C for 12 h to obtain AA@Tf-Lip.

One milliliter of liposome suspension was centrifuged at 3000×g for 5 min at 4 °C. The supernatant was added to a 100 kDa ultrafiltration tube, supplemented with PBS, and centrifuged at 4000×g for 30 min at 4 °C to collect the filtrate (free drugs). The retentate was lysed with 1 mL methanol, centrifuged, and the supernatant (encapsulated drugs) was collected. Standard curves of 5-Aza and ADM were established; free and encapsulated drug concentrations were determined via UV spectrophotometry and fluorescence spectrophotometry, respectively. BCA method was used to quantify the Tf binding efficiency of AA@Tf-Lip. Encapsulation efficiency (EE) and drug loading (DL) were calculated as follows:


$$EE(\%)=\frac{Mass\;of\;encapsulated\;drug\;(mg)}{Mass\;of\;drug\;added\;during\;preparation\;(mg)}\times 100\%$$

$$DL(\%)=\frac{Actual\;mass\;of\;drug\;in\;liposomes\;(mg)}{Actual\;mass\;of\;drug\;in\;liposomes\;(mg)\;+\;Mass\;of\;lipid\;materials\;(mg)}\times 100\%$$

Stability characterization of AA@Tf-Lip: (1) Serum stability: AA@Tf-Lip was incubated with 10% FBS, and the particle size and PDI were detected at different time points (0, 2, 4, 6, 12, 24, 36 h); (2) Storage stability: AA@Tf-Lip was stored at 4°C, and the particle size and PDI were detected at different days (0, 1, 2, 3, 4, 5, 6, 7 days). Dynamic light scattering (DLS) was used to detect the particle size, PDI and zeta potential of three batches of AA@Tf-Lip for statistical analysis.

#### 2.3.7 Drug release assay

Drug-loaded liposomes (1 mL) were placed into a dialysis bag (MWCO 10 kDa) and immersed in 20 mL pH 7.4 PBS (simulating blood) or pH 5.5 PBS (simulating lysosomes) at 37 °C with constant shaking (100 rpm). At predetermined time points (0, 1, 2, 4, 8, 12, 24 h), 1 mL of release medium was sampled (equal volume of fresh buffer was supplemented each time). ADM was detected via fluorescence spectrophotometry (excitation/emission: 470/590 nm), and 5-Aza was detected via UV spectrophotometry (241 nm) to analyze drug release kinetics under different pH conditions.

#### 2.3.8 Cellular uptake assay

HepG2 or HUVEC cells in the logarithmic growth phase were seeded into confocal dishes and allowed to adhere. Cells were divided into three groups: blank control (PBS), free ADM, and AA@Tf-Lip (ADM: 1 μM, 5-Aza: 3 μM). After incubation at 37 °C for 1, 2, 4, 8, and 12 h, the culture was terminated. Cells were washed 3 times with pre-cooled PBS, fixed with 4% paraformaldehyde for 15 min, stained with DAPI for 5 min, and mounted with anti-fluorescence quenching medium. Images were captured immediately via CLSM (excitation: 470 nm, emission: 590 nm) to analyze the distribution intensity and time-dependent differences of ADM in the cytoplasm and nucleus.

#### 2.3.9 Apoptosis assay

Cells in the logarithmic growth phase were seeded into 6-well plates and treated with 1 μM ADM for 24 h. Cells were collected (adherent cells: trypsin-digested; suspended cells: direct centrifugation), washed with PBS, and stained with Annexin V-FITC and PI in the dark at RT for 15 min. Apoptosis was detected via flow cytometry (FITC channel: Annexin V; PI channel: necrotic cells). The proportions of early apoptotic (Annexin V^+^/PI^-^) and late apoptotic (Annexin V^+^/PI^+^) cells were analyzed.

#### 2.3.10 Anti-tumor efficacy assay

HepG2 cells in the logarithmic growth phase were digested, resuspended in PBS containing 50% Matrigel at 1×10^7^ cells/mL, and subcutaneously injected into the right axilla of BALB/c nude mice (4–6 weeks old, 100 μL/mouse). When the tumor volume reached 100–150 mm³, mice were randomly divided into groups (*n* = 5). The experimental group received intravenous injection of AA@Tf-Lip (containing 5 mg/kg ADM, once every 3 days), while control groups received equal volume of PBS or free drugs. Tumor length/width and body weight were recorded every 2 days. At the end of the 15-day experiment, mice were anesthetized; tumors were dissected, weighed, and fixed with 4% paraformaldehyde for HE/TUNEL staining.

#### 2.3.11 *In vivo* survival analysis

Tumor-bearing nude mice were randomly divided into control groups (PBS) and treatment group (AA@Tf-Lip, 5 mg/kg ADM), with intravenous injection once every 3 days (*n* ≥ 10/group). From the first administration day (Day 0), mice were observed daily to record the time of death or reaching the termination criteria. Survival curves were plotted.

#### 2.3.12 Intratumoral drug distribution

HepG2 cells were prepared and injected into nude mice as described in Section 2.3.10. When the tumor volume reached 100–150 mm³, free ADM or AA@Tf-Lip was intravenously injected. After 24 h, tumors were excised, and ADM fluorescence distribution was observed via frozen sectioning.

## 3 Results and discussion

### 3.1 RDM1 expression in HCC and its clinical value as a prognostic biomarker

**Figure 1:**
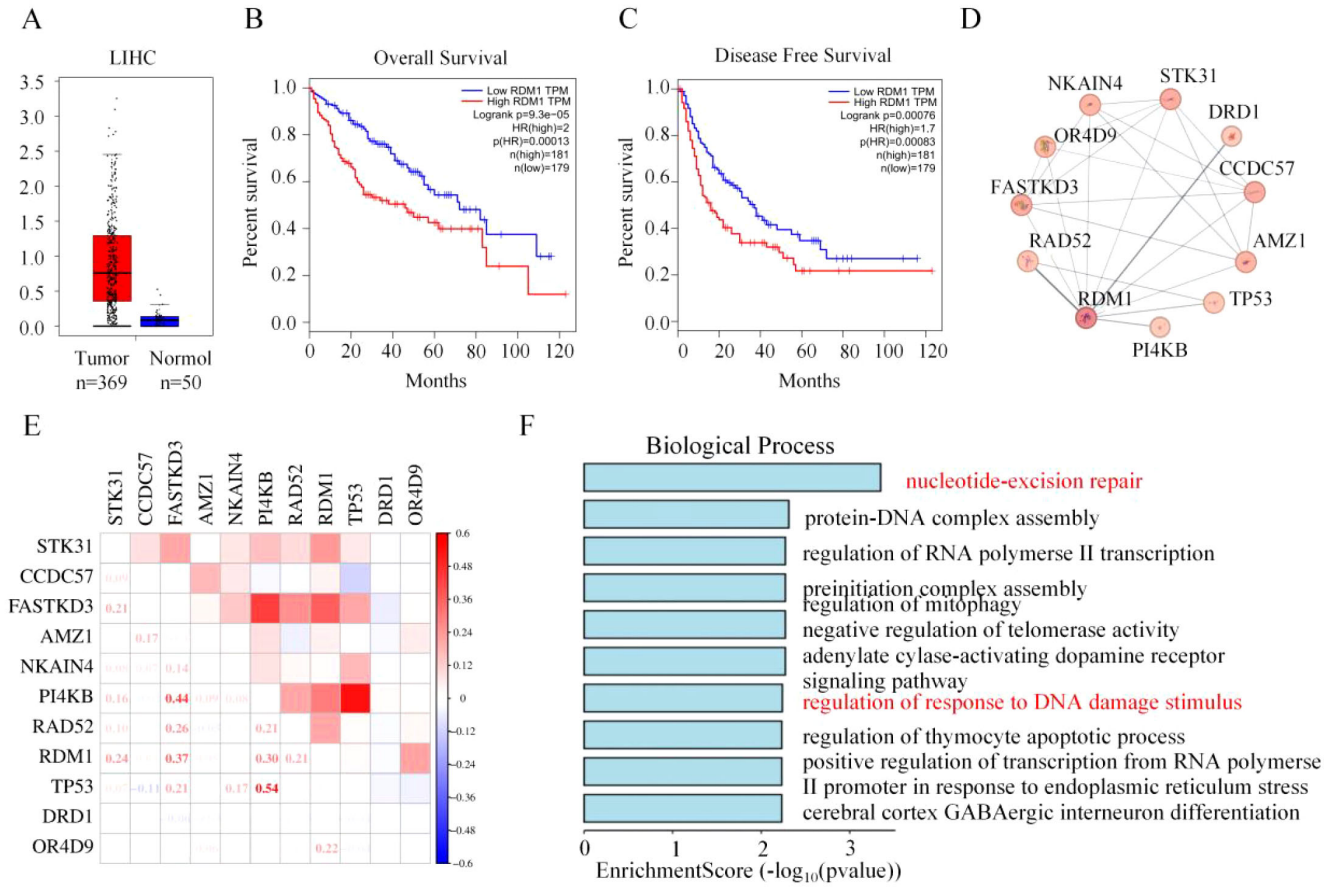
Expression Characteristics of RDM1 in Hepatocellular Carcinoma and Its Clinical Relevance as a Prognostic Biomarker. **(A)** RDM1 expression levels in hepatocellular carcinoma tissues and adjacent non-tumor tissues. **(B)** Overall survival based on RDM1 expression levels in hepatocellular carcinoma. **(C)** Disease-free survival based on RDM1 expression levels in hepatocellular carcinoma. **(D)** Proteins closely interacting with RDM1. **(E)** Correlation between RDM1 and interacting proteins in hepatocellular carcinoma. **(F)** GO (Biological Process) analysis of RDM1 and its interacting proteins.

By integrating TCGA-LIHC clinical samples (16), gene co-expression networks, and *in vitro* experiments, this study clarified the critical role of RDM1 in HCC chemosensitivity regulation. First, significantly increased RDM1 expression was detected in 369 HCC tissues (*p* < 0.001), indicating abnormal activation of RDM1 in tumors (**Figure 1A**). Survival analysis showed that high RDM1 expression was associated with significantly shorter OS and DFS (Log-rank test: *p* < 0.001; HR: 2.0 and 1.7, respectively), confirming RDM1 as an independent poor prognostic predictor for HCC (**Figures 1B, C**).

To explore the molecular mechanism of RDM1, we constructed an RDM1-related gene co-expression network based on Pearson correlation coefficients. As shown in **Figures 1D**, core nodes include STK31, CCDC57, and FASTKD3, whose expression is significantly positively correlated with RDM1, suggesting synergistic effects in the same regulatory pathway. The correlation matrix revealed high expression correlation between RDM1 and key genes such as PI4KB (17) and TP53 (18), implying their joint involvement in tumor proliferation, apoptosis, and signal transduction (**Figure 1E**). GO enrichment analysis showed that RDM1-related genes are significantly enriched in DNA repair processes (e.g., nucleotide excision repair, DNA damage response) (19), as well as RNA polymerase II transcription regulation and mitophagy (20, 21) (**Figure 1F**). These results suggest that RDM1 promotes HCC progression by regulating DNA repair, transcriptional activity, and mitochondrial homeostasis.

In summary, RDM1 is highly expressed in HCC and negatively correlated with patient survival. It mediates multiple biological processes (DNA repair, transcriptional regulation, mitochondrial function) by interacting with PI4KB and TP53, thereby driving the malignant phenotype of HCC. This molecular network provides clues for exploring RDM1 as a therapeutic target and lays a foundation for developing RDM1-targeted chemosensitization strategies.

### 3.2 Synergistic killing of HCC cells by 5-Aza and ADM via RDM1 inhibition

**Figure 2:**
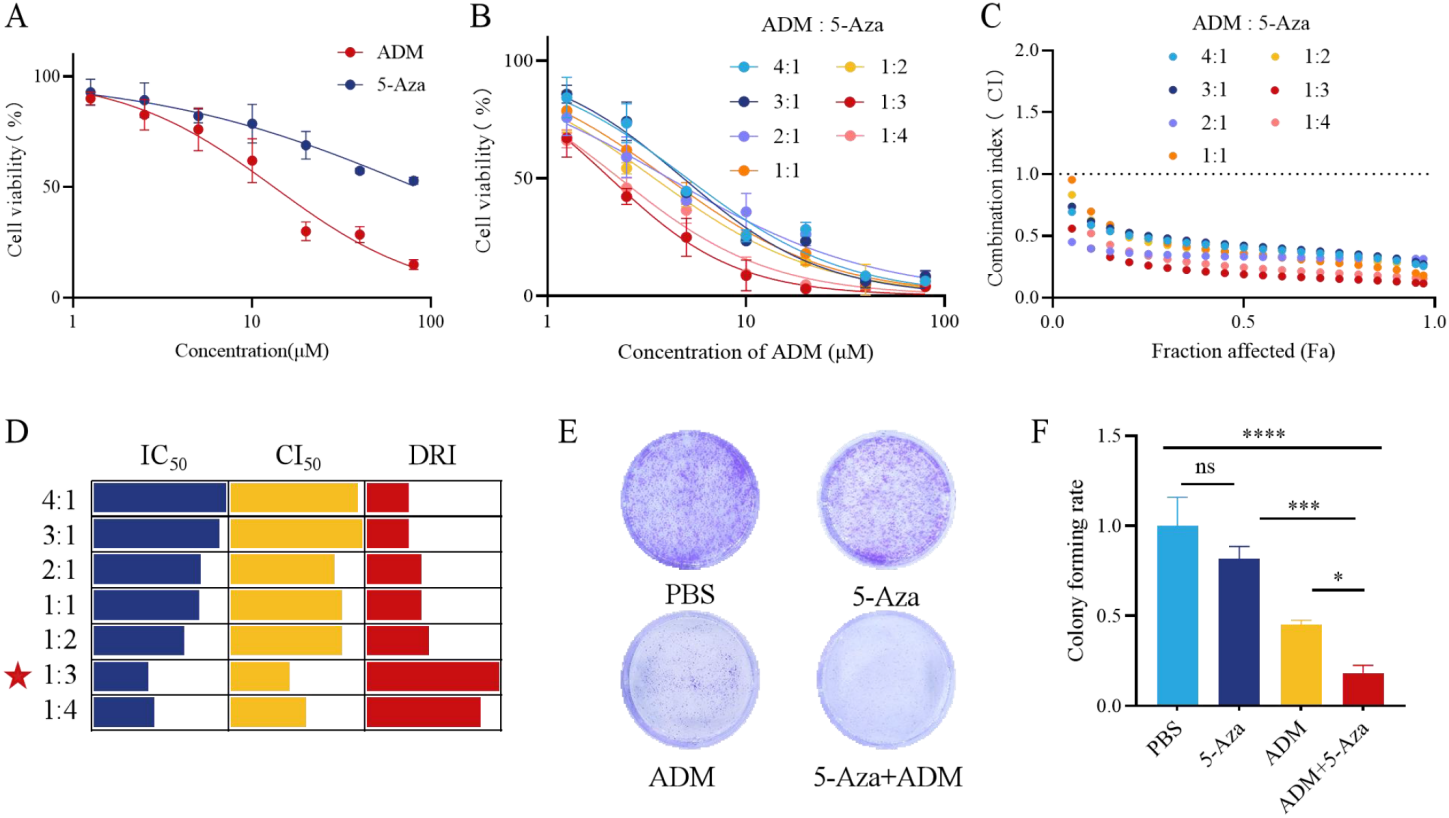
Indirect Inhibition of RDM1 by 5-Aza Synergistically Enhances Hepatocellular Carcinoma Cell Killing with ADM. **(A)** Cytotoxicity of ADM and 5-Aza in HepG2 cells. **(B)** Cytotoxicity of different ratio combinations of ADM and 5-Aza in HepG2 cells. **(C)** Synergistic curves of different ratio combinations of ADM and 5-Aza in HepG2 cells.CI < 1 indicates synergistic effect. **(D)** Changes in IC50, CI50 and DRI of different ratio combinations of ADM and 5-Aza in HepG2 cells. **(E)** Sensitizing effect of 5-Aza on ADM-induced killing of HepG2 cells. **(F)** Quantitative analysis of colony formation assay. Statistical *p*-values: *p < 0.05; ***p < 0.001; ****p < 0.0001; *ns*, no significance.

Based on the critical role of RDM1 in HCC chemosensitivity, we proposed a strategy to enhance chemotherapy sensitivity by inhibiting RDM1. Treating HepG2 cells with 5-Aza (to downregulate RDM1) combined with ADM resulted in significant synergistic cytotoxicity. As shown in **Figure 2A**, cell viability of single-drug treatment decreased in a concentration-dependent manner, and the combined group showed significantly lower survival rate than single-drug groups (**Figure 2B**). CI curves (**Figure 2C**) showed CI < 1 in most dose combinations, confirming synergistic effects. Quantitative analysis (**Figure 2D**) revealed the minimum CI at ADM:5-Aza = 1:3, indicating the optimal synergistic ratio.

Colony formation assay further verified these findings. Compared with the PBS control, single use of 5-Aza or ADM inhibited HepG2 colony formation, while the combined treatment showed the strongest inhibitory effect (**Figure 2E**). Quantitative statistics (**Figure 2F**) confirmed that the colony formation rate of the combined group was significantly lower than that of single-drug groups, revalidating the enhanced lethal effect of ADM by 5-Aza. siRNA-mediated RDM1 knockdown in HepG2 cells efficiently reduced RDM1 mRNA and protein expression by 55.0% ± 4.1% and 52.0% ± 4.1%, respectively; RDM1 knockdown alone significantly sensitized cells to ADM (IC50 from 22.14 μM to 5.11 μM), and the combination of siRDM1 and 5-Aza did not further reduce the IC50 (5.73 μM), confirming that 5-Aza’s chemosensitizing effect is predominantly dependent on RDM1 inhibition (**Supplementary Figure 2**). The chemosensitizing effect of 5-Aza combined with ADM was further validated in Huh7 cells: the combination treatment led to a 60.2% ± 1.5% reduction in cellular viability (**Supplementary Figure 5**), a significant decrease in colony formation efficiency, and a 4.1% ± 0.2% increase in apoptotic rate compared with ADM monotherapy, showing consistent trends with HepG2 cells.

In summary, 5-Aza downregulates RDM1 by reducing the stability of RDM1 mRNA through post-transcriptional mechanisms, impairing the HRR capacity of HCC cells; ADM further amplifies the cell death signal by inducing DSBs. The optimal synergistic effect is achieved at ADM:5-Aza = 1:3, providing a new strategy for HCC combination chemosensitization (22).

### 3.3 Mechanism of RDM1-mediated DNA repair inhibition in chemotherapy sensitization

**Figure 3:**
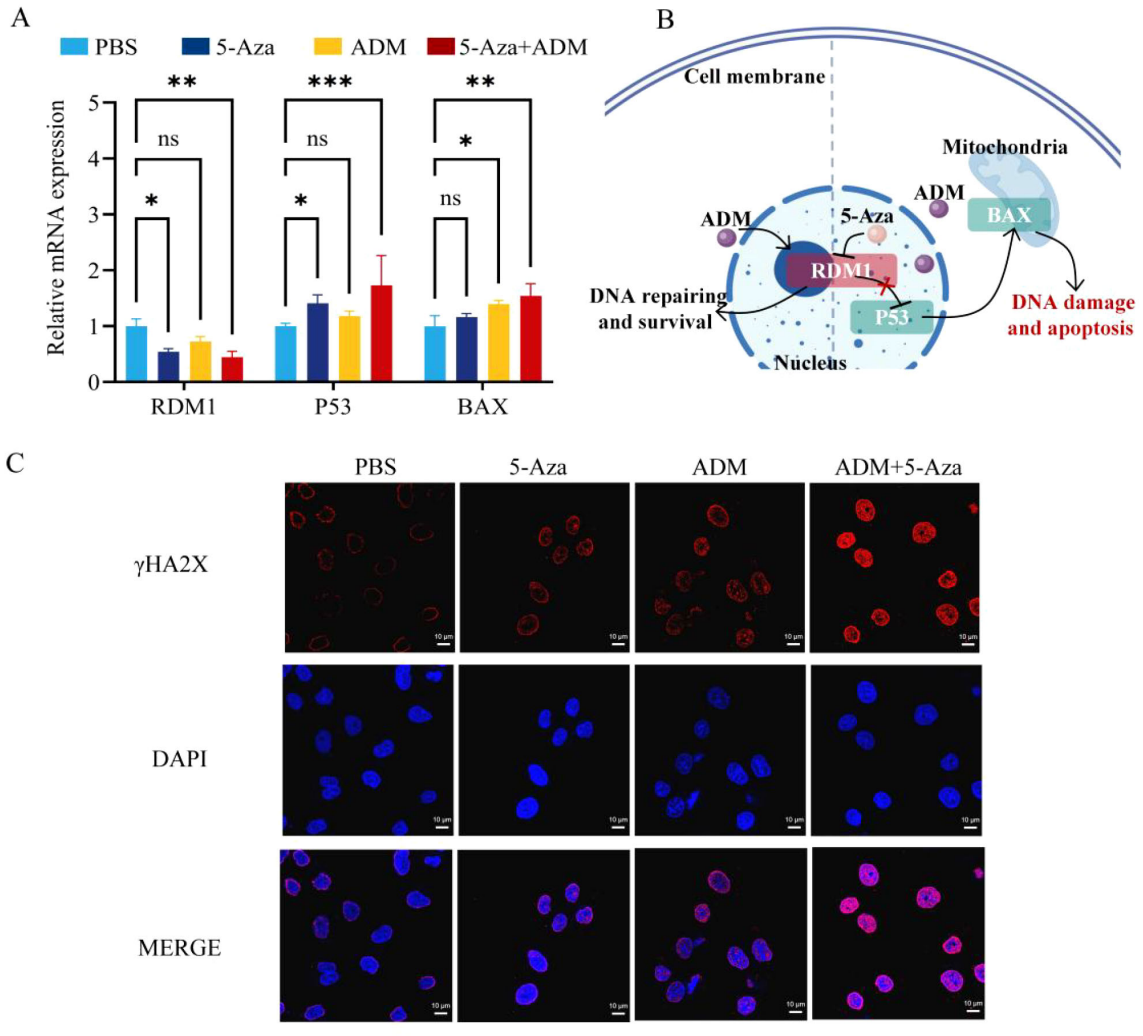
Mechanistic Investigation into Chemosensitization through Inhibition of RDM1-Mediated DNA Repair Pathway. **(A)** mRNA expression levels of RDM1, P53 and BAX, measured by qPCR assay and quantitative analysis. Data are presented as mean ± SD (*n* = 3). **(B)** Schematic illustration of the proposed mechanism (25). **(C)** Immunofluorescence staining for γH2AX. Scale bar: 20 μm.Statistical *p*-values: *p < 0.05; **p < 0.01; ***p < 0.001; *ns*, no significance.

To explore the mechanism of RDM1-mediated DNA repair inhibition in chemotherapy sensitization, we integrated molecular analysis and functional experiments. The PCR results revealed distinct patterns of gene - expression changes across different treatment groups. When compared with the PBS group, the 5 - Aza group exhibited a notable decrease in the mRNA expression level of the RDM1 gene. Simultaneously, the relative mRNA expression of P53 was significantly enhanced in this group. In the ADM group, there was also an observable increase in the mRNA level of BAX. Intriguingly, the 5 - Aza + ADM group presented an even more striking phenomenon: the reduction in the RDM1 mRNA expression level was far more pronounced compared to the 5 - Aza group alone. Moreover, the relative mRNA expression levels of both P53 and BAX in the 5 - Aza + ADM group reached the highest values among all the treatment groups. These findings not only confirm the individual effects of 5 - Aza and ADM on specific gene expressions but also highlight the synergistic impact of their combination on modulating the expression of RDM1, P53, and BAX. (**Figure 3A**). This suggests that RDM1 inhibition enhances apoptotic signals by activating the p53-Bax pathway (23), thereby sensitizing HCC cells to ADM (**Figure 3B**). mRNA stability assay confirmed that 5-Aza treatment significantly accelerated the degradation of RDM1 mRNA and shortened its half-life in HepG2 cells, providing direct evidence that 5-Aza downregulates RDM1 via post-transcriptional mechanisms (**Supplementary Figure 1**). γH2AX immunofluorescence staining showed significantly higher γH2AX focus density in the combined group than in the ADM single-drug and control groups, while 5-Aza alone caused a slight increase (**Figure 3C**). ImageJ quantitative analysis confirmed that the fluorescence intensity of γH2AX in the 5-Aza + ADM group was 9.97 ± 0.61-fold higher than that in the control group (p<0.01) (**Supplementary Figure 6**). This indicates that 5-Aza inhibits RDM1-mediated HRR, leading to unrepaired DNA damage accumulation and exacerbating chemotherapy-induced cell death (24). RAD51 foci formation assay showed that the number of RAD51 foci in the 5-Aza + ADM group was significantly reduced compared with the ADM monotherapy group, directly confirming that RDM1 inhibition impairs the HRR capacity of HCC cells by reducing RAD51 foci formation (**Supplementary Figure 4**).

### 3.4 Design and characterization of AA@Tf-Lip

**Figure 4:**
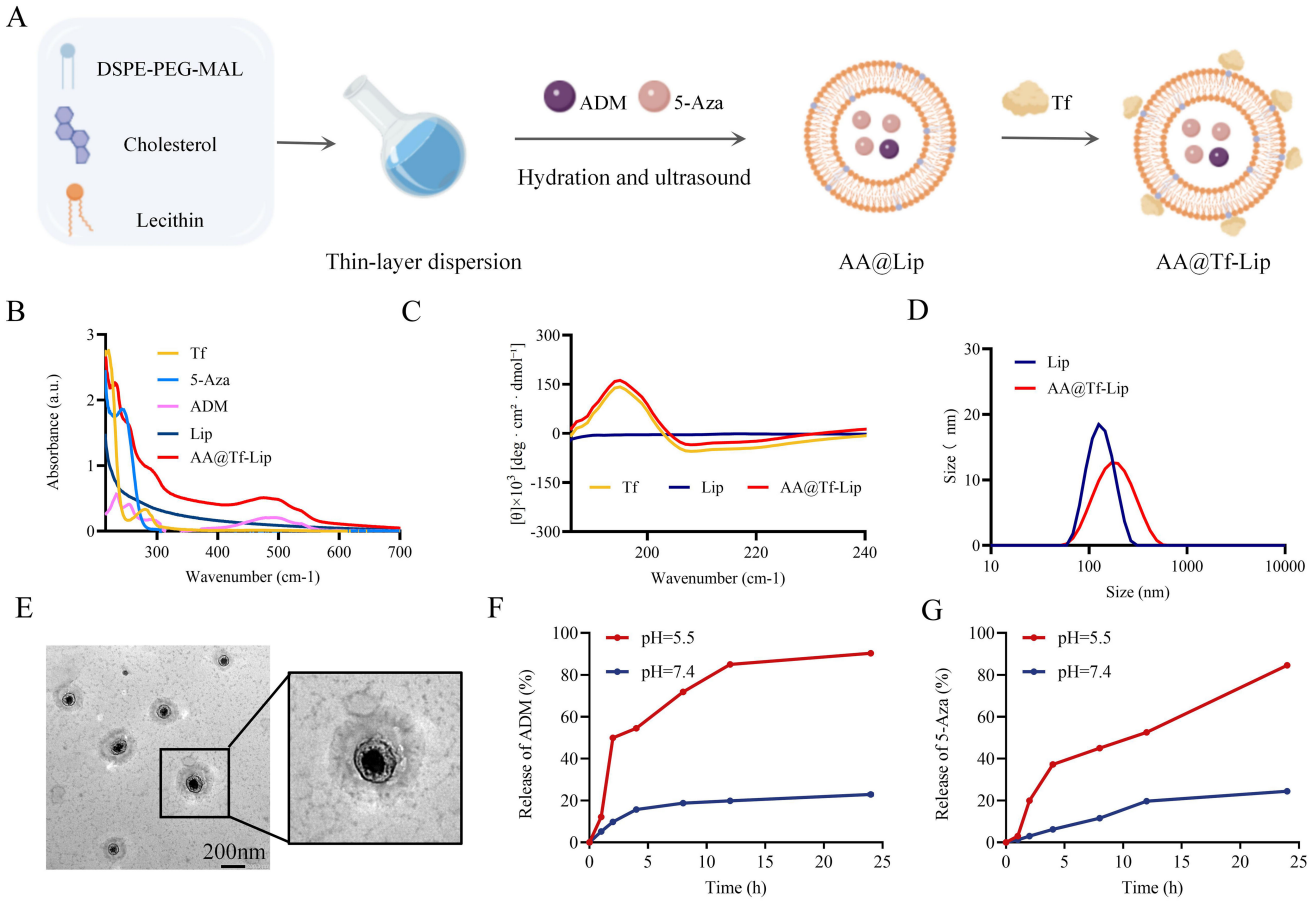
Structural Design and Physicochemical Characterization of AA@Tf-Lip Dual-Drug Nanodelivery System. **(A)** Schematic illustration of AA@Tf-Lip preparation. **(B)** UV-Vis absorption spectra. **(C)** Circular dichroism spectra. **(D)** Dynamic size distribution. **(E)** Transmission electron microscopy (TEM) images. Scale bar: 200 nm. **(F, G)***In vitro* drug release profiles of ADM and 5-Aza in pH 7.4 and pH 5.5 conditions at 37 °C. Data are presented as mean ± SD (*n* = 3).

Although 5-Aza + ADM shows synergistic chemosensitizing effects, their *in vivo* distribution and toxicity limit clinical application. To deliver drugs to tumors at the optimal ratio (26), we designed AA@Tf-Lip (**Figure 4A**). This system encapsulates 5-Aza and ADM via Tf conjugation on liposomes. UV spectroscopy confirmed successful Tf grafting and efficient drug loading at a molar ratio of 1:3 (**Figure 4B**). Circular dichroism spectroscopy showed intact secondary structure of Tf post-conjugation, ensuring TfR binding activity (**Figure 4C**). Dynamic light scattering (DLS) detection of three batches of AA@Tf-Lip showed a particle size of 153.05 ± 3.15 nm, PDI = 0.23, zeta potential = -21.41 ± 0.35 mV; BCA method quantified the Tf binding efficiency as 47.84% ± 0.65% (**Figures 4D, E**, **Supplementary Table 2**). The EE was 67.8% (both ADM and 5-Aza) and DL of 5.57% (ADM) and 7.51% (5-Aza). Under pH 5.5 (lysosomal simulation), ADM release reached 71.87% at 8 h and 84.96% at 12 h (**Figure 4F**), while 5-Aza release exceeded 80% at 24 h (**Figure 4G**). Stability tests showed that AA@Tf-Lip had good serum stability (particle size variation <10% over 36 h, PDI maintained at 0.15-0.22) and storage stability (physicochemical properties changed <15% after 7 days at 4°C) (**Supplementary Figure 3**). The two drugs reach therapeutic concentrations simultaneously in tumor cells: ADM induces DSBs, and 5-Aza maintains RDM1 silencing by post-transcriptional regulation, blocking HRR. This achieves synergistic sensitization and reduces normal tissue exposure, verifying AA@Tf-Lip as a precise HCC delivery platform.

### 3.5 Targeting and safety of AA@Tf-Lip

**Figure 5:**
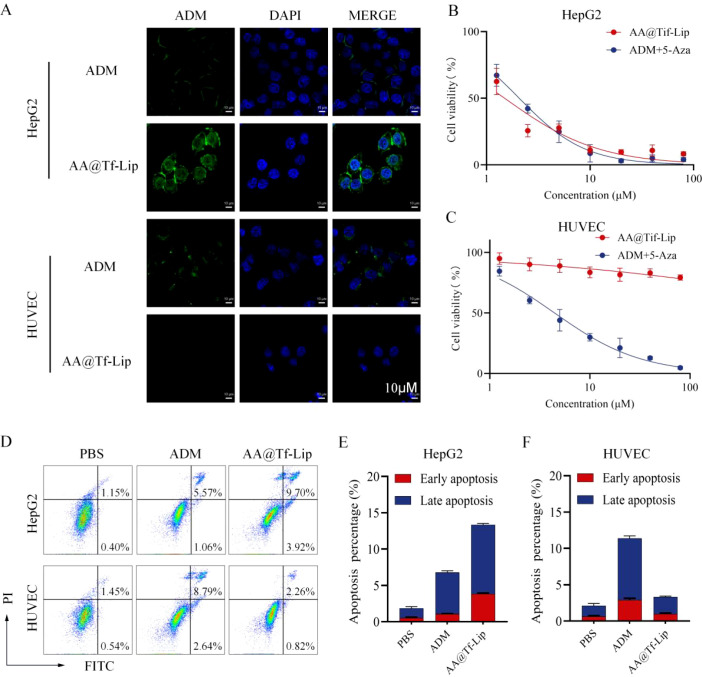
Targeting and Safety of AA@Tf-Lip Dual-Drug Delivery System. **(A)** Cellular uptake of ADM in HepG2 and HUVEC cells. Scale bar: 10μm. **(B)** Cytotoxicity in HepG2 cells. Data are presented as mean ± SD (*n* = 3). **(C)** Cytotoxicity in HUVEC cells. Data are presented as mean ± SD (*n* = 3). **(D)** Flow cytometry analysis of apoptosis showing Annexin V-FITC and PI staining in HepG2 and HUVEC cells after treatment with PBS, free ADM, and AA@Tf-Lip. **(E, F)** Quantitative analysis of apoptosis in HepG2 cells and HUVEC cells. Data are presented as mean ± SD (*n* = 3).

*In vitro* experiments verified the targeting and selective cytotoxicity of AA@Tf-Lip. AA@Tf-Lip achieves specific targeting of HepG2 via Tf-mediated receptor endocytosis (27): compared with free ADM, AA@Tf-Lip shows significantly higher uptake in HepG2, while no significant difference was observed in HUVEC (**Figure 5A**), confirming Tf-mediated selective enrichment in TfR-high tumor cells.

Cytotoxicity (**Figures 5B, C**) and apoptosis (**Figures 5D–F**) experiments further confirmed this: in HepG2, AA@Tf-Lip reduced IC_50_ from 2.07 μM to 1.79 μM and increased apoptosis rate by 1.97-fold compared with free drugs; in HUVEC, free drugs showed an IC_50_ of 4.44 μM, while AA@Tf-Lip showed almost no cytotoxicity and reduced apoptosis rate by 3.42-fold. These results confirm the good biocompatibility and safety of AA@Tf-Lip, providing a reliable platform for precise HCC chemosensitization treatment.

### 3.6 Anti-tumor efficacy of AA@Tf-Lip in nude mouse models

**Figure 6:**
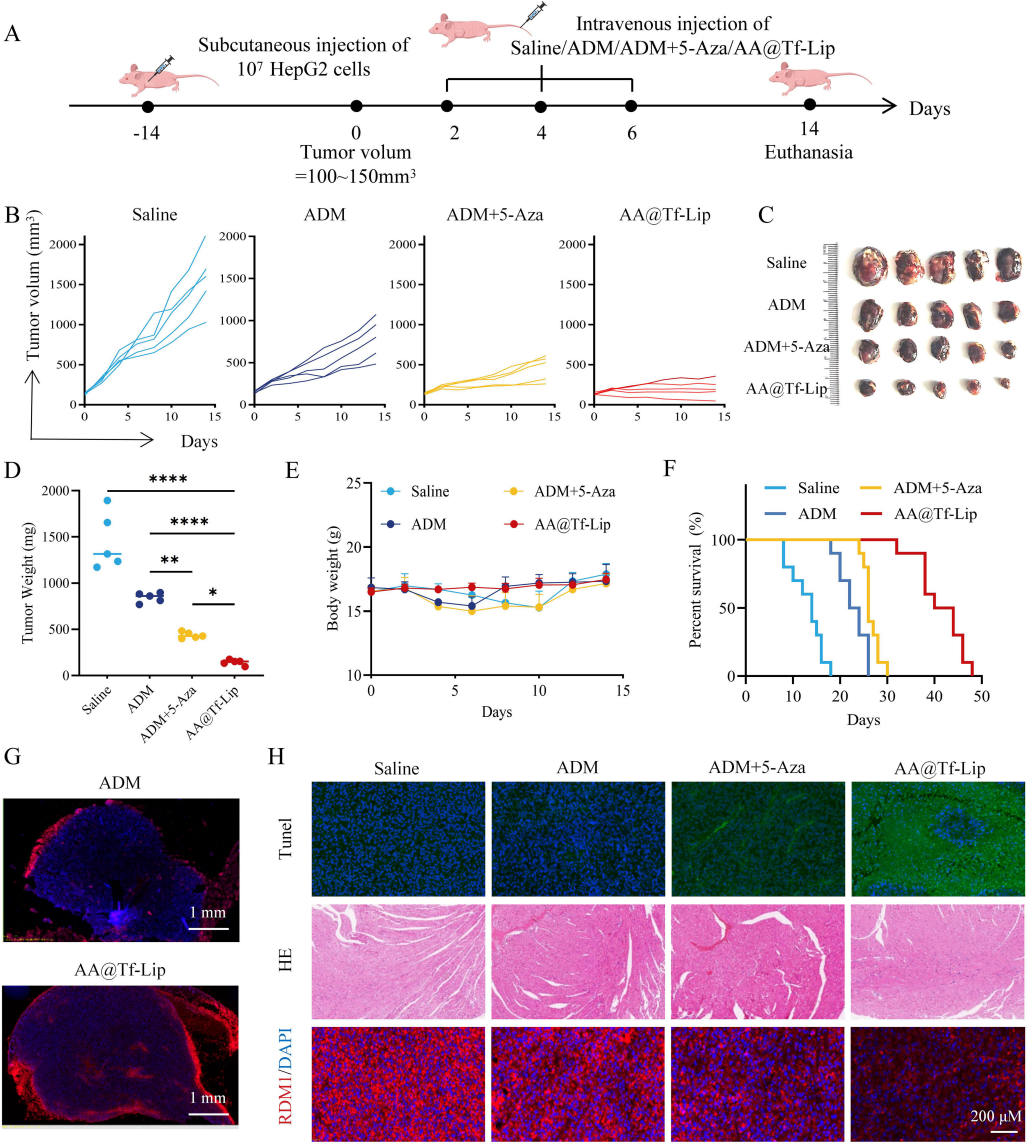
Antitumor Efficacy of AA@Tf-Lip Dual-Drug Nanodelivery System in Hepatocellular Carcinoma Xenograft Model. **(A)** Schematic diagram showing the establishment of HepG2 subcutaneous xenograft model in BALB/c nude mice and the treatment schedule. **(B)** Tumor volume changes over time in different treatment groups. Data are presented as mean ± SD (*n* = 5). **(C)** Photographs of tumors excised from mice in different treatment groups at the end of the experiment (Day 15). **(D)** tumor weights in different treatment groups at the end of the experiment. Data are presented as mean ± SD (*n* = 5). **(E)** Body weight changes of mice in different treatment groups during the experimental period. **(F)** Survival curves of tumor-bearing mice in different treatment groups. **(G)** Tumor distribution of ADM. ADM fluorescence is shown in red. Scale bar: 1 mm. **(H)** TUNEL, H&E, and RDM1 immunofluorescence staining of tumor tissues from different treatment groups. Scale bar: 200 μm. Statistical *p*-values: *p < 0.05; **p < 0.01; ****p < 0.0001; *ns*, no significance.

The therapeutic efficacy of AA@Tf-Lip was further evaluated in a BALB/c nude mouse subcutaneous model of hepatocellular carcinoma (HCC) (**Figures 6A, B**). Compared with free ADM or the combination of ADM and 5-Aza, treatment with AA@Tf-Lip resulted in significantly superior tumor growth inhibition. By day 15, the tumor suppression rate in the AA@Tf-Lip group was markedly higher than that in all other treatment groups. These findings were corroborated by consistent trends observed in tumor photographs (**Figure 6C**) and tumor weights (**Figure 6D**). As shown in **Figure 6E**, AA@Tf-Lip group showed no weight loss, while the free drug group showed significant weight loss, indicating reduced off-target toxicity of AA@Tf-Lip. Survival curves (**Figure 6F**) showed significantly prolonged median survival in the AA@Tf-Lip group, while no significant difference was observed between ADM single-drug and saline groups, and the ADM + 5-Aza group showed limited improvement. This confirms that AA@Tf-Lip prolongs survival by reducing tumor burden and systemic toxicity.

Intratumoral ADM distribution (**Figure 6G**) showed that free ADM fluorescence was limited to the tumor surface, while AA@Tf-Lip fluorescence penetrated into the tumor interior, confirming enhanced tumor drug uptake. Histological analysis (**Figure 6H**) showed higher apoptotic cell proportion in the AA@Tf-Lip group via TUNEL staining, and significantly reduced RDM1 expression via immunofluorescence. HE staining of heart tissue showed neatly arranged myocardial fibers in the AA@Tf-Lip group (similar to the saline group), while ADM and ADM + 5-Aza groups showed irregular arrangement and cell pyknosis/karyolysis. These results confirm that AA@Tf-Lip efficiently inhibits tumor growth, enhances apoptosis, and reduces systemic toxicity, verifying its feasibility as a precise HCC chemosensitization carrier.

## 4 Conclusion

RDM1 is a multifunctional oncogene that helps HCC cells reduce chemosensitivity and drives proliferation. To address RDM1-mediated low chemosensitivity in HCC, we constructed AA@Tf-Lip, a dual-drug delivery system targeting HCC cells for co-delivering ADM and 5-Aza. In this strategy, 5-Aza downregulates RDM1 by reducing the stability of RDM1 mRNA through post-transcriptional mechanisms, impairing HRR capacity; ADM enhances DSBs to amplify apoptotic signals. Tf-mediated active targeting improves drug enrichment and penetration in tumors and reduces normal tissue exposure. The chemosensitizing effect of this strategy was validated in both HepG2 and Huh7 cell lines with distinct genetic backgrounds, and the core mechanism was confirmed by genetic validation experiments of RDM1 knockdown. *In vivo* experiments confirmed that AA@Tf-Lip inhibits tumor growth, prolongs survival, and reduces ADM-related cardiotoxicity. However, This study employed a subcutaneous xenograft model to validate therapeutic efficacy, which has certain limitations: (1) It cannot fully replicate the hepatic microenvironment of HCC; (2) It lacks the natural characteristics of tumor angiogenesis, which may affect the tumor penetration efficiency of nanocarriers. Future research will adopt an *in situ* hepatocellular carcinoma model to more closely approximate the pathological features of clinical HCC. Despite these limitations, the core mechanism (RDM1-mediated chemosensitization) and the targeting capability of the delivery system have been sufficiently validated, laying the foundation for subsequent studies (28). In conclusion, AA@Tf-Lip achieves RDM1-mediated chemotherapy sensitization and overcomes off-target toxicity, providing a precise solution for improving chemosensitivity in HCC.

## Data Availability

The raw data supporting the conclusions of this article will be made available by the authors, without undue reservation.
