# Supplementary material for: Transferrin-modified liposomes enhance chemosensitivity in hepatocellular carcinoma by suppressing RDM1-mediated DNA repair
Source: Front Oncol. 2026 Mar 23;16:1792167. doi: 10.3389/fonc.2026.1792167 (PMC13051216; doi:10.3389/fonc.2026.1792167)
Supplement: Supplementary file 1 [file DataSheet1.docx]

**Transferrin-Modified Liposomes Enhance Chemosensitivity in Hepatocellular Carcinoma by Suppressing RDM1-Mediated DNA Repair**

Xiaoni Cai^a,b,1^, Xiang Wang^c,d,1^, Qiongdan Zhang^e,f,1^, Fan Gao^a,b^, Song Xu^a,b^, Wenqiao Lyu^a,b^, Jiayuan Ye^g,b^, Fei Liu^h,i,j,*^, Luting Zhang^a,b,**^

**^a^** *Department of General Surgery, Shangyu People's Hospital of Shaoxing, Shaoxing, Zhejiang 312300, China*

**^b^** *School of Medicine, Shaoxing University, Shaoxing, Zhejiang 312000, China*

**^c^** *Department of Hepatobiliary Surgery, Shandong Provincial Third Hospital, Shandong University, Jinan, Shandong 250031, China*

**^d^** *Shandong Provincial Third Hospital Medical Research Center, Hepatobiliary Minimally Invasive Research Lab, Jinan, Shandong 250031, China*

**^e^** *Department of The First Clinical Medical School**, Hainan Medical University, Haikou, Hainan 571199, China*

**^f^** *Hainan Academy of Medicine Sciences, Haikou, Hainan 571199, China*

**^g^** *Department of Infectious Diseases, Shangyu People's Hospital of Shaoxing, Shaoxing, Zhejiang 312300, China*

**^h^** *Department of Hepatobiliary Surgery, The First People’s Hospital of Lianyungang, Lianyungang, Jiangsu 222002, China*

**^i^** *Lianyungang Clinical College of Nanjing Medical University, Lianyungang, Jiangsu 222002, China*

**^j^** *The First Affiliated Hospital of Kangda College of Nanjing Medical University, Lianyungang, Jiangsu 222002, China*

**^*^** Corresponding author at: *The First People’s Hospital of Lianyungang, No.182 Tongguan North Road, Haizhou District, Lianyungang, Jiangsu 222002, China.*

**^**^** Corresponding author at: *Shangyu People's Hospital of Shaoxing, No.517 Minzhu Road, Bai Guan Street, Shangyu District, Shaoxing, Zhejiang 312300, China*

*E-mail* *addresses:* feiliu1226@163.com (F. Liu), surgeonzhang1016@163.com (L. Zhang).

^1^ Xiaoni Cai, Xiang Wang and Qiongdan Zhang contributed equally to this work.

**Experimental Procedures**

1. **mRNA stability assay:** Cells were divided into a control group and a 5-Aza-treated group (3 μM for 24 hours). Both groups were then co-treated with the transcriptional inhibitor actinomycin D (5 μg/mL) to block new mRNA synthesis. Cell samples were collected at 0, 2, 4, 6, and 8 hours post-inhibition, and total RNA was extracted and reverse-transcribed into cDNA. Real-time quantitative PCR was performed to detect the relative expression levels of RDM1 mRNA at different time points. Finally, the degradation rate constant k was calculated using the half-life formula t1/2=ln2/k based on the mRNA decay curve over time, and the half-life was determined. Differences between the two groups were compared to assess whether 5-Aza promotes the degradation of RDM1 mRNA.

2. **RDM1 siRNA Knockdown Experiment:** HepG2 cells were seeded in 6-well plates and transfected with RDM1-specific siRNA (si-RDM1) or negative control siRNA (NC) using Lipofectamine 3000 according to the manufacturer's instructions. After 48 hours of transfection, the knockdown efficiency of RDM1 mRNA and protein was verified by qPCR and Western blot, respectively. Subsequently, the effects of RDM1 knockdown on ADM sensitivity were assessed by CCK-8 assay and flow cytometry apoptosis assay.

3. **AA@Tf-Lip Characterization of Physicochemical Properties (Particle Size, Zeta Potential, Transferrin Binding Efficiency, and Stability):** Three batches of AA@Tf-Lip (n=3) were prepared. An aliquot of 100 μL of the suspension was diluted to a concentration of 100 μg/mL, and the particle size distribution, polydispersity index (PDI), and zeta potential were measured using dynamic light scattering (25°C, 90° scattering angle). The binding efficiency of transferrin (Tf) was determined by the BCA method: First, 1 mg of liposomes incubated with transferrin were thoroughly washed to remove unbound free Tf. Subsequently, the liposomes were lysed to release the bound Tf, and the concentration of Tf protein in the supernatant was quantified by the BCA method, followed by calculation of the mass of bound Tf. Finally, the transferrin binding efficiency was expressed as the percentage of the mass of bound Tf relative to the total mass of liposomes (1 mg). The specific calculation formula was: Transferrin binding efficiency (%) = (Mass of Tf measured by BCA / Total mass of liposomes involved in the reaction) × 100%.

4. **RAD51 Immunofluorescence Staining**: HepG2 cells were inoculated onto confocal culture dishes (pre-treated with coversLip) and divided into four groups: PBS control, 5-Aza group (3 μM, 24 h), ADM group (1 μM, 24 h), and 5-Aza+ADM group (1 μM ADM co-cultured with 3 μM 5-Aza for 24 h after 6 h 5-Aza pretreatment). Each group consisted of 3 replicates. After treatment, immunofluorescence staining was performed: 4% polyformaldehyde fixation, 0.1% Triton X-100 permeabilization, 5% BSA blocking, followed by rabbit anti-human RAD51 primary antibody (1:200) incubated overnight at 4°C. Goat anti-rabbit secondary antibody labeled with Alexa Fluor 488 (1:500) was incubated at room temperature in the dark for 1 h. DAPI was used for nuclear counterstaining, and slides were sealed with anti-fluorescence quencher. Imaging was performed under a laser confocal microscope. The average number of focal points per cell nucleus was statistically analyzed using ImageJ software.

1. ****Verification of the effects of RDM1-related processing on chemosensitivity, proliferation, and apoptosis in Huh7 cells：**Logarithmic growth phase Huh7 cells (cultured in DMEM with 10% FBS, sourced from ATCC) were used to replicate the core in vitro experiments with HepG2 cells. The experimental groups included: PBS control, ADM (1 μM), and AA@Tf-Lip. Each group consisted of 3 replicates. Cell viability was assessed using the CCK-8 assay (ADM concentration: 1 μM; 5-Aza concentration: 3 μM). Colony formation was evaluated after 10 days of treatment by counting colonies and calculating colony formation rate. Apoptosis rates were determined by flow cytometry. Results from Huh7 cells were compared with HepG2 cells through consistency analysis to validate the robustness of conclusions across cell lines.**


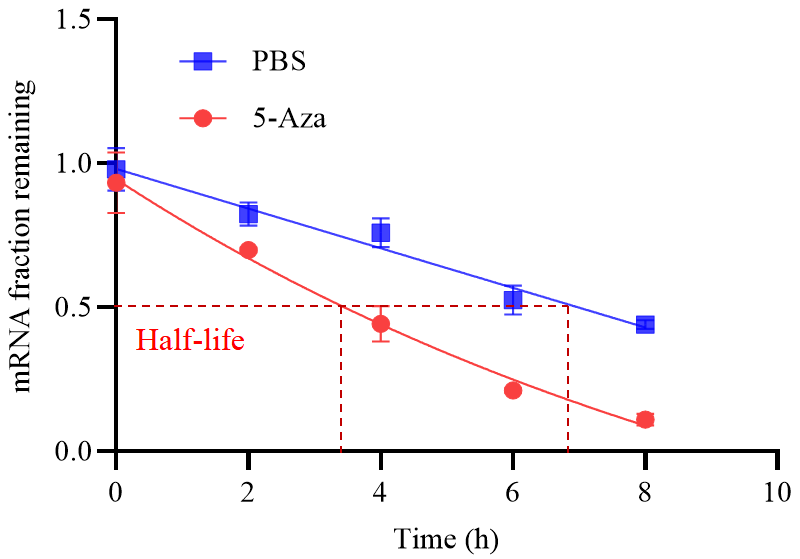


**Figure S1.** qRT-PCR anaiysis for the expression of RDM1 mRNA after different treatments, data presented as mean ± SD (n=3).


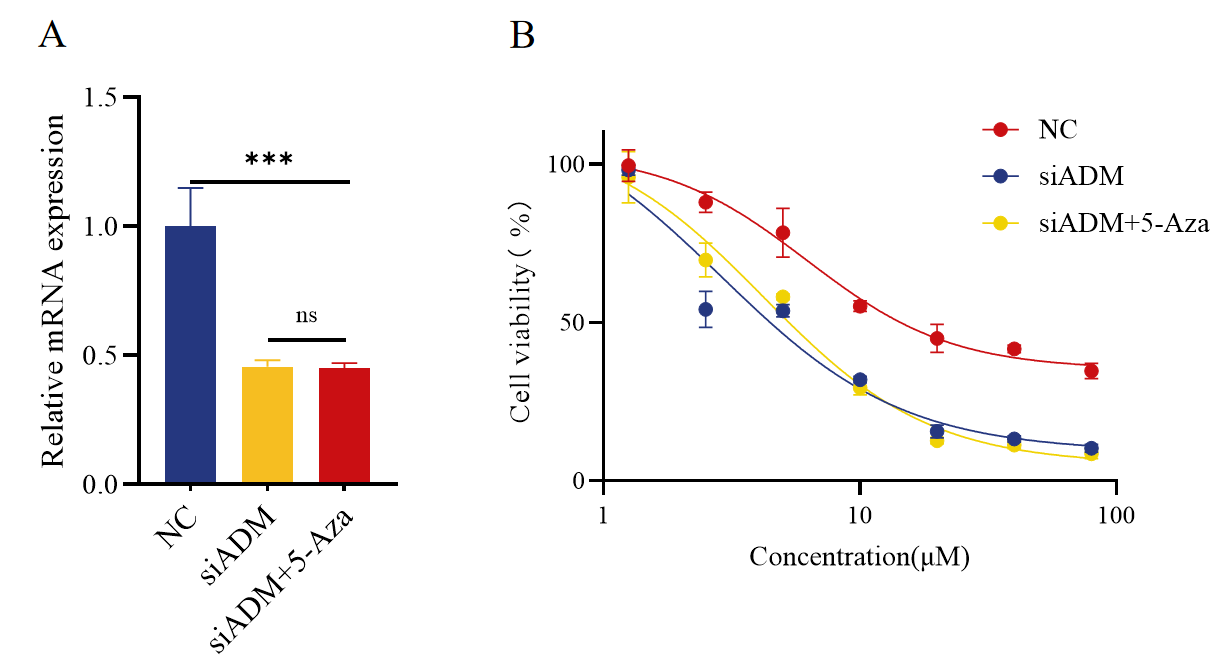


**Figure S2. Effects of RDM1 knockdown on ADM chemosensitivity in HepG2 cells** (A) qPCR analysis of relative RDM1 mRNA expression levels in cells across groups. (B) CCK-8 assay to evaluate cell viability after 24-hour treatment with ADM at different concentrations, data presented as mean ± SD (n=3).

**Figure S3**. (A) Stability testing at 4°C. (B) Stability testing with 10% FBS serum.


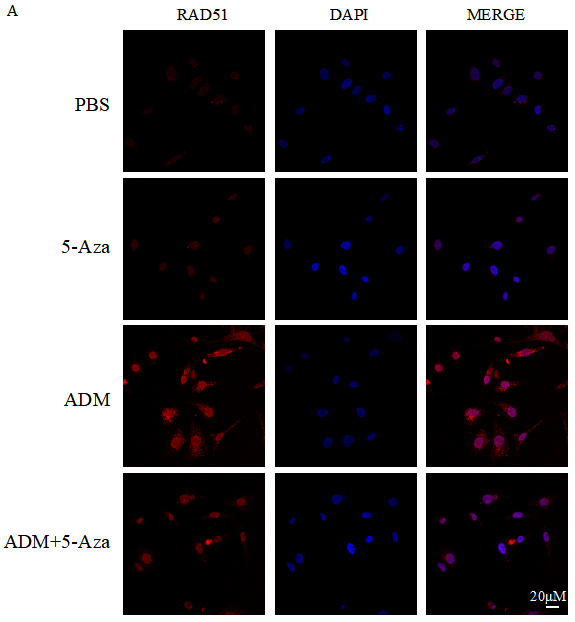


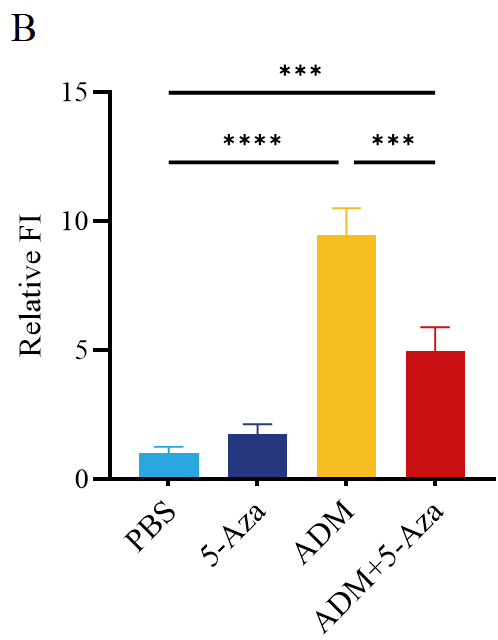


**Figure S4. **Immunofluorescence detection of the effects of different treatments on RAD51 formation in HepG2 cells**. (A) Representative laser confocal images. Green fluorescence (Alexa Fluor 488) labeled RAD51 protein, blue fluorescence (DAPI) labeled cell nuclei. Scale bar = 10 μm. (B) RAD51 fluorescence quantification，** data presented as mean ± SD (n=3).


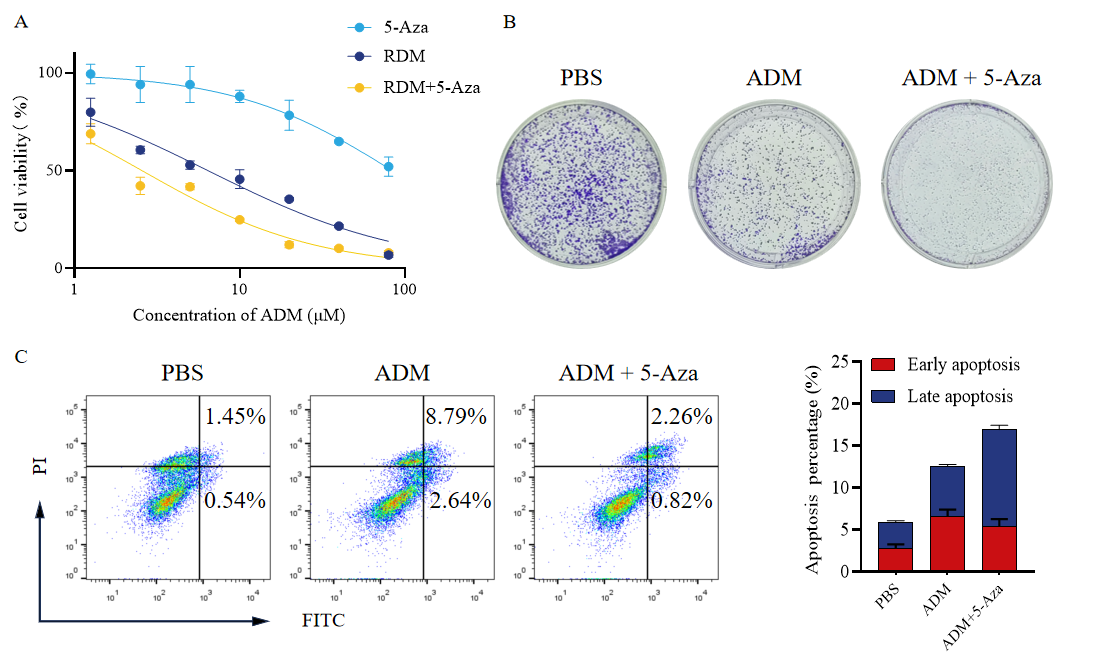


**Figure S5. **Verification of the effects of RDM1-related treatments on chemosensitivity, proliferation, and apoptosis in Huh7 cells.** (A) CCK-8 assay to determine cell viability in Huh7 cells treated with different agents (ADM 1 μM, 5-Aza 3 μM). (B) Representative images and quantitative statistics of colony formation assays. (C) Representative scatter plots and apoptosis rate statistics (Annexin V-FITC/PI double staining) obtained by flow cytometry. Data are expressed as mean ± SD (n=3).**


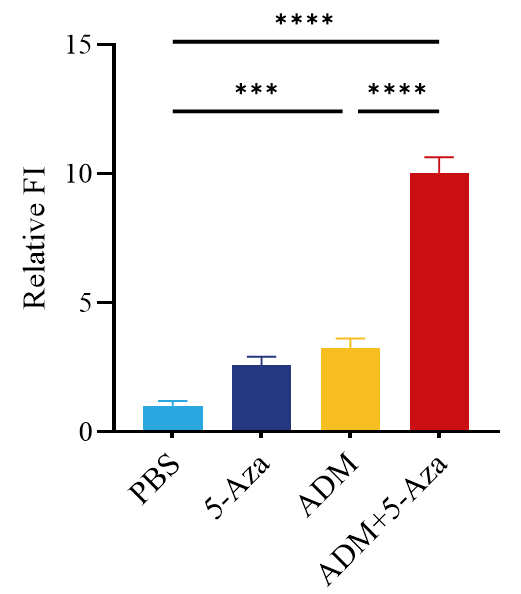


**Figure S6.**quantitative analysis of γH2AX by immunofluorescence(n = 3)

**Table S1**.Primer sequences for qPCR

| ***Gene*** | **Forward sequence (5'-3')** | **Reverse sequence (5'-3')** |
| --- | --- | --- |
| *GAPDH* | TCTCTGCTCCTCCCTGTTC | ACACCGACCTTCACCATCT |
| *RDM1* | GCCCATCCTGGTTTCTATGCC | AGACGAACCTTGACTGGAGAT |
| *TP53* | TGTAACAGTTCCTGCATGGGC | GACAGGCACAAACACGCACC |
| *BAX* | GACCCGGTGCCTCAGGATGC | AGGTCAGCTCATCATGCTTG |

**Table S2.** Particle size, zeta potential and transferrin binding efficiency of AA@Tf-Lip. (n = 3)

**Table S3**. the specific p-values

Table S4. Abbreviation Table

| Abbreviation | Full Name |
| --- | --- |
| RDM1 | RAD52 motif-containing 1 |
| DSBs | DNA double-strand breaks |
| HCC | Hepatocellular Carcinoma |
| ADM | Doxorubicin |
| 5-Aza | 5-azacytidine |
| Tf | Transferrin |
| TfR | Transferrin receptor |
| HRR | Homologous recombination repair |
| MSP | Methylation-specific PCR |
| DLS | Dynamic light scattering |
| PDI | Polydispersity Index |
| qPCR | Quantitative Polymerase Chain Reaction |
| CI | Combination index |
| EE | Encapsulation efficiency |
| DL | Drug loading |
| OS | Overall survival |
| PFS | Progression-free survival |
| DFS | Disease-free survival |
| RM-ANOVA | Repeated measures analysis of variance |
